# Impact of health workforce availability on health care seeking behavior of patients with diabetes mellitus in China

**DOI:** 10.1186/s12939-017-0576-0

**Published:** 2017-07-01

**Authors:** Yinzi Jin, Weiming Zhu, Beibei Yuan, Qingyue Meng

**Affiliations:** China Center for Health Development Studies, Peking University, 38 Xue Yuan Road, Haidian District, Beijing, 100191 China

**Keywords:** Health workforce, Outpatient visits, Choice of health providers, Diabetes mellitus patients

## Abstract

**Background:**

China has a high burden of diabetes mellitus (DM), and a large proportion of DM patients remain untreated for various reasons, including low availability of primary health care providers. DM patient management is one of the priorities in China’s national essential public health programs. Shortage of health workforce has been a major barrier to improving access to health care for DM patients. This study examines the impact of the health workforce on outpatient utilization of DM patients.

**Methods:**

Data were collected from China National Health Service Surveys in 2008 and 2013, covering 94 rural counties and 156 urban districts, respectively, with a total of 15,984 DM patients. Household data and facility-based data at county/district level were merged. The health workforce was measured by number of physicians per 1,000 population in county hospitals and primary health centers (PHCs), respectively. Health care seeking behavior was measured by health care utilization and distribution of health providers of the DM patients. Multilevel zero-inflated negative binomial regression was used to analyze the impact of the health workforce on outpatient visits by DM patients, and a multilevel, multinomial logit model was used to examine the impact of the health workforce on choice of health providers by DM patients.

**Results:**

An increase in the number of physicians at both county hospitals and PHCs was associated with increased outpatient visits by DM patients, particularly more physicians at PHCs. With increased numbers of physicians at PHCs, outpatient visits among residents with DM in rural and western areas of China increased more than those in urban and eastern areas. More physicians at PHCs had a positive impact on improving the likelihood of outpatient visits at PHCs. The positive influence of increasing the number of physicians available to DM patients in rural and western areas was greater than that for urban and eastern DM patients.

**Conclusions:**

The health workforce is a key component of any healthcare system and is critical in improving health care accessibility. Strategies to increase coverage of health workforce at PHCs are crucial to achieving adequate levels of health services for DM patients. Allocation of health workforce should focus on PHCs in rural and low-income areas.

## Background

China has the highest number of people with diabetes mellitus (DM) in the world, accounting for 25% of total DM patients in the globe in 2013 [1]. Prevalence of DM increased from 0.9% in 1990 to 9.7% in 2008, and 11.6% in 2013 [2–4]. DM raises the risk of developing long-term cardiovascular disease and other complications and represents a fast-growing disease burden and considerable economic consequences for individuals, communities, and health systems [5, 6]. Timely and effective use of health care, particularly primary health care, is essential to reduce the disease burden of DM [7]. Adequate human resources for health are needed to provide accessible and sustainable health care for DM patients in China [8]. Even though effective therapies and treatment guidelines for managing diabetes have been readily available, only 30% of people with DM in China have been diagnosed, 25% of those have received treatment, 40% of which have maintained glycemic control [4]. Those living in rural and less-developed regions have more constraints in access to health care [9].

China’s health system reform in 2009 pays attentions to public health care through an essential public health program that is fully funded by the government [10]. Government-funded primary health centers (PHCs), village clinics and township health centers in rural counties and community health centers (stations) in urban cities, are the main providers for delivering a package of public health services. Public health activities for DM care include four visits to PHCs each year by the DM patients and referral to specialist care in hospitals by PHCs if necessary [11, 12]. PHCs have been regarded as critical health providers for disease prevention and control [13]. The Chinese government expanded the coverage of social health insurance and raised premium subsidies to cover at least 50% of expenditures for outpatient services in 2015 [14]. To improve the capacity of the primary health workforce, the government implemented a number of measures, including support education and training programs for increasing availability of health workforce for rural and PHCs [15].

China faces shortage and maldistribution of health workforce. These challenges are more serious in rural areas and less-developed western and central regions [16]. It has been difficult to recruit and retain health workers at PHCs due to low income and limited professional development opportunities in PHCs. Distribution of qualified health workforce concentrates in urban cities and hospitals in China. The density of doctors in urban cities was twice that in rural areas in 2013, and qualification of health professionals in high-income regions is much higher than that in less-developed regions [17].

Little is known about the impact of availability of health workers on health care accessibility. Studies on health care seeking behavior in China focus on analysis of individual and household factors without inclusion of health system factors such as health workforce [18, 19], even though some studies qualitatively examines the association of availability of health workers and access to health care [20–22]. Several international studies examine the relationship between increase in health workers and changes in health outcomes using econometrics [23–28], but what is the relationship between availability of health workers and health care seeking behaviors is not examined. Health care seeking behavior measured by health care utilization and choice of health providers, is an important proxy for health outcome. This study aims to examine the relationship between availability and distribution of health workforce and health care seeking behavior using data from China’s national health service surveys.

## Methods

### Data source

This study used two databases. One is China National Health Service Survey (NHSS) conducted in 2008 and 2013, and the other is the routine reporting data on health resources. The NHSS is a nationally representative, repeated cross-sectional survey that has been conducted every 5 years since 1993. This study used data of waves of 2008 and 2013. The NHSS uses a multistage stratified random cluster sampling method to sample county and county-level districts in each of 31 sampled provinces. In each county or district, five sample townships or communities are selected; then two villages or neighborhoods in each selected township or community are randomly selected. Within each selected village or neighborhood, 60 households are randomly selected. All residents in the sampled households are surveyed using a standard questionnaire for their demographics, health status, health behaviors, and health care use. All residents aged 15 years or older are asked to report whether they have been diagnosed with diabetes.

The routine reporting data on health resources include number and distribution of health institutions and facilities, number and distributions of health workforce, and healthcare expenditures.

In our study, we inserted the variables of per capita GDP (PGDP) at county level, the number of physicians per 1,000 population at PHCs, and physicians per 1,000 population at hospitals into the NHSS household data in 2008 and 2013. In total, 177,501 and 273,687 individuals were surveyed in 94 and 156 NHSS counties/districts in 2008 and 2013, respectively, which included 15,984 self-reported DM patients aged 15 years or older in 2008 and 2013.

### Measures

We measured the county-level density of health workforce in each county using the number of physicians per 1,000 population at county hospitals, and the number of physicians per 1,000 population at PHCs. We measured health care seeking behavior with the number of outpatient visits by DM　patients　and the type of health providers visited during the previous 2 weeks when the survey was conducted. Types of health providers included village clinics, township health centers, community health stations and centers, county hospitals, and municipal or provincial hospitals, which are mutually exclusive.

DM patients were categorized based on residence locations of rural or urban, and eastern or central or western. Urban area and eastern region are more economically developed than rural and other regions. The prevalence of DM was age-standardized with national census data of 2010. The impact of health workforce availability on use of outpatient service by DM patients was estimated by two dimensions, including the impact of physician density on outpatient visits by DM patients and the impact of physician distribution at county hospitals and PHCs on choice of the health providers.

### Statistical analysis

We used outpatient visits by DM patient as dependent variable, and physician density in PHCs or hospitals in each county as major independent variable. To account for unmeasured variations within each county, we used multilevel random intercept analyses to process the two-level structure of individual-level and county-level data.

First, given the data type of the dependent variables and the number of outpatient visits with extra zeros and over-dispersion, multilevel zero-inflated negative binomial regression (ML_ZINB) was applied to analyze the impact of physician density on outpatient visits by patients with DM.

Let y be the number of outpatient visits, the ML_ZINB distribution of outpatient visits can be written as:

$$ \begin{array}{l} P\left({\pi}_{ij}=0\right)={\pi}_{ij}+\left(1-{\pi}_{ij}\right){\left(1+\upalpha {\pi}_{ij}\right)}^{a^{-1}}\\ {} p\left({\pi}_{ij}= k\right)=\left(1-{\pi}_{ij}\right)\frac{\varPi \left( k+{a}^{-1}\right)}{k!\varPi \left({a}^{-1}\right)}{\left[\frac{a^{-1}}{a^{-1}+{\lambda}_{ij}}\right]}^{a^{-1}}{\left[\frac{\lambda_{ij}}{a^{-1}+{\lambda}_{ij}}\right]}^k,\cdot k>0\end{array} $$

where 0 < π < 1, and π is the probability of an extra zero response, λ is the mean, and α is the dispersion parameter of the underlying NB distribution.

Let *y*_*ij*_ (i = 1, 2, …, m; j = 1, 2, …, n) be the count of outpatient visits by *j*th DM patients in *i*th county. In our study, DM patients are nested in counties, and outpatient visits by DM patients belonging to different counties are independent, whereas they are correlated for those who live in the same county. NB models for counts permit λ to depend on the explanatory variables. Then, linear predictors *τ*_*ij*_ and *φ*_*ij*_ are defined as:

$$ \begin{array}{l} \log \kern0.5em  it\left({\pi}_{i j}\right)={\tau}_{i j}=\gamma {w}_{i j}+{\delta}_i\\ {} \log \left({\lambda}_{i j}\right)={\varphi}_{i j}=\beta {x}_{i j}+{\varepsilon}_i\end{array} $$

where covariates *w*_*ij*_ and *x*_*ij*_ appearing in the logistic and NB components are not necessarily the same. Vectors *δ*_*i*_ and *ε*_*i*_ denote county-specific random impacts.

Second, a multilevel multinomial logit model (MML) was used to examine the impact of the distribution of physicians at county hospitals and PHCs on the choice of health providers, with the type of health provider as the dependent variable.

Let y denote the probability of the choice of health providers. The MML is a mixed generalized linear model with linear predictors:

$$ {\eta}_{i j}^{(m)}={\alpha}^{(m)}+{\beta}^{(m)}{x}_{i j}+{\xi}_i^{(m)}+{\delta}_{i j}^{(m)} $$

and multinomial logit link:

$$ p\left({Y}_{i j}= m\left|{x}_{i j},{\xi}_i,{\delta}_{i j}\right.\right)=\frac{ \exp \left\{{\eta}_{i j}^{(m)}\right\}}{1+{\displaystyle {\sum}_{l=2}^M \exp \left\{{\eta}_{i j}^{(1)}\right\}}} $$

where m = 1, 2, …, M denotes the choice of health providers, i = 1, 2, …, l denotes the county, and j = 1, 2, …, J denotes DM patients in *i*th county. The response variable *y*_*ij*_ has a multinomial distribution (conditional on the random impacts), taking values in the set of categories {1, 2, …, M}.

The linear predictors have specific parameters *a* ^(*m*)^ and *β* ^(*m*)^ (m = 1, 2, …, M). Finally, *ξ*_*i*_ and *δ*_*ij*_ are vectors of random errors representing unobserved heterogeneity at county level and individual level, respectively, with the following distributional assumptions. Errors at different levels are independent:

$$ \begin{array}{l}{\xi}_i^{\hbox{'}}=\left({\xi}_i^{(2)},\dots, {\xi}_i^{(M)}\right)\hbox{'}\frac{ i id}{\sim } N\left(0,{\displaystyle \sum \xi}\right)\\ {}{\delta}_{i j}^{\hbox{'}}=\left({\delta}_{i j}^{(2)},\dots, {\delta}_{i j}^{(M)}\right)\hbox{'}\frac{ i id}{\sim } N\left(0,{\displaystyle \sum \delta}\right)\end{array} $$

An alternative specification of the multinomial logit model is based on the random utility model. If the continuous random Variables *U*_*ij*_^(*m*)^, m = 1, 2, …, and M represent the individual utilities associated to the M choice, the utility maximization rule implies that the observed indicator *y*_*ij*_ equals m only if *U*_*ij*_^(*m*)^ ⋅ > ⋅ *U*_*ij*_^(1)^.

Generally, variances in use of outpatient service were estimated at both county and individual levels in all multilevel regressions. The variance size at both county and individual levels was presented relative to the overall variance. Impact sizes as the result of the ML_ZINB were presented as incidence rate ratio (IRR) with the corresponding 95% confidence interval (CI), whereas impact sizes of the mixed multinomial logit regressions were expressed as relative risk ratio (RRR) with the corresponding 95% CI.

### Control variables

This analysis controls for variables that may influence the relationship between health workforce and use of outpatient service among DM patients, based on existing empirical studies [28–30]. Given the availability of data, the controlled factors in our study are divided into four components, including predisposing factors (age, sex, marital status, education, occupation), enabling factors (income, health insurance status, distance to the nearest healthcare provider), health needs (sickbed days for the illness, presence of other chronic diseases), and environmental indicators (residence location, time period).

Following the analysis in terms of impact size, comparisons of impact were conducted between rural and urban areas as well as between western, central, and eastern regions. The western region include 12 provinces of Sichuan, Qinghai, Xinjiang and others. The eastern region include 11 provinces of Beijing, Tianjin, Zhejiang and others. The central region include 8 provinces of Shanxi, Anhui, Henan and others. All statistical analyses were conducted using Stata 13.1 (StataCorp LP, College Station, TX, USA).

## Results

### Characteristics and health care utilization of the DM patients

Outpatient visits during the previous 2 weeks and percentage of visits to PHCs were compared among urban/rural areas and eastern/central/western regions in 2008 and 2013 (Table 1). The mean rate of outpatient visits within the previous 2 weeks was 12.5% in 2013, less than that in 2008 at 14.3%. The mean rate of outpatient visits of urban residents was higher than that of rural residents in both 2008 (15.1% versus 12.3%) and 2013 (13.2% versus 11.3%). Regional variations in outpatient visits existed in both 2008 and 2013. In 2013, 14.9% of people with diabetes had outpatient visits within the previous 2 weeks in eastern regions, more than in central (9.7%) and western (12.8%) regions, 66.3% of the DM patients choosing PHCs. Higher proportion of rural patients used PHCs than urban patients in both 2008 (74.5 vs 44.5%) and 2013 (72.9% vs 52.7%); same trends were found between the three regions.

**Table 1:** Prevalence and outpatient use by patients with diabetes mellitus in 2008 and 2013

	2008			2013		
	Urban	Rural	Total	Urban	Rural	Total
Age-standardized prevalence (mean‰ [mean ± SD])						
All	27.5 (26.6, 28.4)	4.8 (4.5, 5.1)	10.7 (10.1, 11.3)	49.5 (42.4, 56.6)	21.8 (19.7, 23.9)	35.2 (32.0, 38.4)
East	30.8 (28.5, 33.1)	19.6 (17.4, 21.8)	26.6 (24.5, 28.7)	57.9 (50.7, 65.1)	29.8 (27.6, 32.0)	44.1 (41.3, 26.9)
Central	28.8 (25.6, 32.0)	15.8 (14.3, 17.3)	18.2 (15.6, 20.8)	49.5 (41.6, 57.4)	36.0 (32.4, 39.6)	35.8 (31.6, 40.0)
West	20.8 (18.7, 22.9)	7.8 (7.2, 8.4)	8.7 (7.9, 9.5)	39.5 (35.4, 43.6)	12.7 (9.7, 15.7)	25.1 (21.7, 28.5)
Rate of previous 2-week outpatient visits (mean% [mean ± SD])						
All	15.1 (14.6–15.6)	12.3 (11.8–12.8)	14.3 (13.7–14.9)	13.2 (12.7–13.7)	11.3 (10.8–11.8)	12.5 (12.0–13.0)
East	15.6 (15.0–16.2)	16.3 (15.7–16.9)	15.8 (15.2–16.4)	15.6 (15.0–16.2)	13.8 (13.3–14.3)	14.9 (14.3–15.5)
Central	13.8 (13.2–14.4)	8.6 (8.1–9.1)	12.3 (11.7–12.9)	10.7 (10.3–11.1)	8.2 (7.8–8.6)	9.7 (9.3–10.1)
West	15.4 (14.8–16.0)	10.5 (10.0–11.0)	14.5 (13.9–15.1)	13.3 (12.8–13.8)	11.8 (11.3–12.3)	12.8 (12.3–13.3)
percentage of outpatient visits to PHCs (%)						
All	44.5	74.5	67.3	52.7	72.9	66.3
East	39.3	65.8	48.8	49.6	68.5	55.1
Central	48.8	74.6	53.6	53.6	72.5	67.1
West	56.1	76.6	75.6	60.5	76.7	75.2

The main characteristics of DM patients are reported in Table 2. Around 44 and 46% of patients were rural residents in 2008 and 2013, respectively. The average age decreased from 55.8 years in 2008 to 52.1 years in 2013. Average household income increased from 7,530 RMB in 2008 to 13,210 RMB in 2013. Around half of the DM patients had completed primary school education. In 2008, about 75% of the DM patients were covered by either rural or urban health insurance schemes. The health insurance coverage extended to 85% in 2013.

**Table 2:** Characteristics of the DM respondents

Characteristics (%)	2008	2013
Female	50.17	52.24
Rural residents	44.36	46.53
Age (years) ^a^	55.81(9.16)	52.15(9.22)
Currently Married	75.08	76.37
Household income per capita (1000 RMB) ^a^	7.53(10.49)	13.21(15.17)
Education		
No formal education	15.81	12.36
Primary school	37.65	36.54
Junior and high school	40.71	43.50
Junior college and above	5.83	7.60
Employment status		
Farmers	47.15	45.30
Unemployed or retired	14.27	13.78
Informal employed	11.52	13.25
Formal employed	27.06	27.67
Health insurance status		
New Rural Cooperative Medical Scheme (NRCMS)	57.68	48.74
Urban Employee-based Medical Insurance (UEBMI)	2.90	14.85
Urban Resident-based Medical Insurance (URBMI)	13.69	22.49
Having other chronic diseases	24.85	30.67
Sickbed days for the illness (days) ^a^	0.81 (0.19)	1.02 (0.11)
Distance to the nearest healthcare provider		
Less than 2 km	79.91	80.57%
2–4 km	12.74	13.96%
4- km and farther	7.35	5.47%
Per capita GDP (PGDP) (1000 RMB) ^a^	18.55 (4.27)	40.31 (11.45)
N	1953	14031

^a^Mean (SD)

### Physician density and distribution

Density and distribution of physicians are presented in Table 3. Physician density at PHCs was 0.44 per 1,000 population in 2013, much lower than that in county hospitals at 1.47. The gap of physician density between PHCs and hospitals widened from 2008 to 2013.

**Table 3:** Physician density and distribution in sample counties during 2008 and 2013

	2008			2013		
	Urban	Rural	Total	Urban	Rural	Total
Physicians in PHCs per 1000 people (mean [mean ± SD])						
All	0.46 (0.42–0.50)	0.39 (0.35–0.43)	0.41 (0.36–0.46)	0.56 (0.51–0.61)	0.37 (0.34–0.40)	0.44 (0.40–0.48)
East	0.54 (0.49–0.59)	0.38 (0.35–0.41)	0.44 (0.39–0.49)	0.66 (0.60–0.72)	0.41 (0.36–0.46)	0.51 (0.45–0.57)
Central	0.38(0.34–0.42)	0.43 (0.39–0.47)	0.41 (0.36–0.46)	0.46 (0.41–0.51)	0.38 (0.34–0.42)	0.42 (0.38–0.46)
West	0.37 (0.33–0.41)	0.38 (0.34–0.42)	0.39 (0.35–0.43)	0.61 (0.56–0.66)	0.32 (0.28–0.36)	0.40 (0.35–0.45)
Physicians in hospitals per 1000 people (mean [mean ± SD])						
All	1.96 (1.89–2.03)	0.55 (0.51–0.59)	0.74 (0.68–0.80)	2.62 (2.49–2.75)	0.80 (0.76–0.84)	1.47 (1.43–1.51)
East	2.11 (2.03–2.19)	0.50 (0.46–0.54)	0.82 (0.76–0.88)	3.56 (3.35–3.77)	0.93 (0.88–0.98)	2.00 (1.94–2.06)
Central	1.64 (1.56–1.72)	0.71 (0.66–0.76)	0.79 (0.73–0.85)	2.33 (2.24–2.42)	0.77 (0.73–0.81)	1.39 (1.33–1.45)
West	2.11 (1.98–2.24)	0.49 (0.45–0.53)	0.65 (0.60–0.70)	1.83 (1.74–1.92)	0.73 (0.69–0.77)	1.07 (1.02–1.12)

In 2013, physician densities at PHCs and hospitals in urban areas were 0.56 and 2.62 per 1,000 population, respectively, higher than that in rural areas with 0.37 and 0.80 per 1,000 population. Eastern region had a higher density of physicians in both PHCs and hospitals than central and western regions in 2013.

### Impact of health workforce on outpatient visits

Result on impact of physician density on outpatient visits by DM patients is reported in Table 4. After adjusting for all potential confounders, as physician density at PHCs increased by 1.00, the mean number of outpatient visits increased by 25% among the total population of DM patients; the size of the positive impact was significantly greater than that for physician density at hospitals (4%). Outpatient visits of DM patients seemed to increase less with an additional physician at PHCs when PGDP of the county is increased, whereas outpatient visits seemed to increase more with an additional physician in hospitals. Physician density in rural areas generally had greater positive impact on outpatient visits than that in urban areas, particularly physician density at PHCs (1.34 versus 1.19). Each physician per 1,000 population added to PHCs in western regions was associated with 67% more outpatient visits, which shows higher correlation than that in in central (40%) and eastern (23%).

**Table 4:** Impact of physician density on outpatient visits among patients with diabetes mellitus

	Model 1		Model 2	
	IRR^*^ (95%CI)	*p* value	IRR^*^ (95%CI)	*p* value
All				
Physicians in PHCs	1.19 (1.02, 1.40)	0.027	1.25 (1.21, 1.29)	<0.001
Physicians in hospitals	1.20 (1.08, 1.35)	<0.001	1.04 (1.01, 1.04)	<0.001
PGDP			1.10 (1.04, 1.15)	<0.001
Physicians in PHCs with PGDP			0.83 (0.73, 0.94)	0.003
Physicians in hospitals with PGDP			1.15 (1.08, 1,24)	<0.001
Rural				
Physicians in PHCs	1.39 (1.29, 1.49)	<0.001	1.34 (1.17, 1.53)	<0.001
Physicians in hospitals	1.02 (1.00, 1.04)	0.062	1.02 (1.01, 1.04)	<0.001
PGDP			1.04 (0.98, 1.10)	0.170
Physicians in PHCs with PGDP			0.73 (0.70, 0.77)	<0.001
Physicians in hospitals with PGDP			0.99 (0.98, 1.02)	0.825
Urban				
Physicians in PHCs	1.13 (1.05, 1.21)	0.001	1.19 (1.15, 1.23)	<0.001
Physicians in hospitals	1.04 (0.93, 1.16)	0.462	1.01 (1.00, 1.02)	0.015
PGDP			1.23 (1.04, 1.46)	0.014
Physicians in PHCs with PGDP			0.98 (0.98, 0.99)	<0.001
Physicians in hospitals with PGDP			1.17 (1.06, 1.30)	0.002
East				
Physicians in PHCs	1.18 (1.01, 1.39)	0.037	1.23 (1.19, 1.28)	<0.001
Physicians in hospitals	1.02 (1.00, 1.04)	0.069	1.02 (1.01, 1.03)	<0.001
PGDP			1.17 (1.03, 1.32)	0.012
Physicians in PHCs with PGDP			0.92 (0.90, 0.95)	<0.001
Physicians in hospitals with PGDP			0.99 (0.97, 1.02)	0.721
Central				
Physicians in PHCs	1.33 (1.16, 1.51)	<0.001	1.40 (1.10, 1.78)	0.005
Physicians in hospitals	1.02 (0.90, 1.16)	0.721	1.04 (1.01–1.07)	0.020
PGDP			1.01 (0.90, 1.12)	0.896
Physicians in PHCs with PGDP			0.87 (0.81, 0.94)	<0.001
Physicians in hospitals with PGDP			1.08 (0.96, 1.22)	0.171
West				
Physicians in PHCs	1.45 (1.06, 1.99)	0.021	1.67 (1.38–2.02)	<0.001
Physicians in hospitals	0.92 (0.56, 1.53)	0.762	0.93 (0.83–1.03)	0.156
PGDP			0.97 (0.86, 1.10)	0.669
Physicians in PHCs with PGDP			0.84 (0.80, 0.88)	<0.001
Physicians in hospitals with PGDP			1.15 (0.99, 1.33)	0.071

* IRR: incidence rate ratio

Model 1. Variables controlled include age, sex, urban/rural residence, marital status, education, occupation, income, health insurance status, presence of other chronic diseases, distance to the nearest healthcare provider, sickbed days for the illness

Model 2. Variables controlled include age, sex, marital status, education, occupation, income, health insurance status, presence of other chronic diseases, distance to the nearest healthcare provider, sickbed days for the illness

### Impact of health workforce on choice of health providers

Analysis on impact of physician density on choice of health providers by DM patients is presented in Table 5. Relative to village clinics, the probability of visiting other PHCs increased 1.14 times if one additional physician per 1,000 population was added in PHCs, whereas the probability of visiting county hospitals decreased by 69%. Relative to village clinics, the odds of choosing other PHCs and county hospitals were 1.15 and 1.31, respectively, if one additional physician was added in the county hospitals.

**Table 5:** Impact of physician distribution on choice of healthcare provider

	PHCs		county/district hospitals		Municipal hospitals and above	
	RRR (95%CI)	*p* value	RRR (95%CI)	*p* value	RRR (95%CI)	*p* value
All						
Physicians in PHCs	2.14 (1.52–3.03)	<0.001	0.31 (0.20–0.48)	<0.001	0.69 (0.32–1.46)	0.331
Physicians in hospitals	1.15 (1.03, 1.28)	0.012	1.31 (1.16, 1.49)	<0.001	1.36 (1.15, 1.60)	<0.001
Rural						
Physicians in PHCs	3.63 (2.64, 4.98)	<0.001	1.68 (1.15, 2.45)	0.007	0.23 (0.13, 0.40)	<0.001
Physicians in hospitals	1.09 (0.94–1.26)	0.256	1.21 (1.06–1.39)	0.005	1.03 (0.90–1.19)	0.625
Urban						
Physicians in PHCs	1.22 (0.93–1.62)	0.152	0.86 (0.65–1.14)	0.207	1.74 (1.34–2.26)	<0.001
Physicians in hospitals	1.21 (1.08–1.36)	0.001	2.22 (1.94–2.53)	<0.001	2.22 (1.78–2.78)	<0.001
East						
Physicians in PHCs	1.83 (1.33–2.53)	<0.001	0.47 (0.32–0.69)	<0.001	0.29 (0.16–0.53)	<0.001
Physicians in hospitals	1.25 (1.14–1.38)	<0.001	1.75 (1.57–1.94)	<0.001	2.12 (1.84–2.45)	<0.001
Central						
Physicians in PHCs	1.80 (0.97–3.37)	0.063	0.78 (0.39–1.58)	0.499	0.03 (0.01–0.11)	<0.001
Physicians in hospitals	1.11 (0.97–1.29)	0.114	1.42 (1.21–1.66)	<0.001	1.09 (0.91–1.30)	0.352
West						
Physicians in PHCs	5.31 (3.57–7.90)	<0.001	4.00 (2.45–6.53)	<0.001	1.92 (0.80–4.60)	0.142
Physicians in hospitals	1.04 (0.85–1.27)	0.720	1.46 (1.16–1.83)	0.001	1.49 (1.11–2.01)	0.008

Increase in physician density at PHCs would reduce the probability of DM patients to choose municipal-level or above hospitals (RRR = 0.69), but an inverse impact of physician density at county hospitals (RRR = 1.36) was found. The impact of physician density at PHCs on the probability of rural DM patients (RRR = 3.63) visiting PHCs was greater than the impact on urban patients (RRR = 1.22). In contrast, urban DM patients were 2.22 times more likely to choose county hospitals relative to village clinics with a higher physician density at county hospitals, that was greater than the probability for rural patients (RRR = 1.21).

The probability of DM patients in the western region choosing PHCs was 5.31 times greater if one additional physician was added in PHCs, which was much higher than that in eastern (RRR = 1.83) and central (RRR = 1.80) regions.

## Discussion

This study examines the relationship between availability of health workforce and use of outpatient service by DM patients by rural and urban area and by region. This study expands the existing studies in terms of its database and statistical methods.

We found that use of outpatient service by DM patients increased and higher level of availability of health workforce in PHCs would lead to higher health care utilization. Over the past decade, China has achieved great success in expanding social health insurance coverage. Demand for health care has been rapidly increasing along with increased affordability of the people [31]. Declining out-of-pocket payments and funding policies for reimbursing patients with chronic diseases including diabetes have contributed to push demands for health care by patients with DM [32]. However, the supply side of health care has not been strengthened in its capacity in meeting the increasing demand [33]. One of the biggest constraints in health care supply side is shortage of health workers, especially in PHCs. Therefore, health care seeking behavior would be sensitive in response to increase in health workforce. Our finding is consistent with existing studies showing the positive association between number of health workers and health care use [21, 25, 34].

This study shows that the impact of physician density at PHCs on outpatient visits was greater than this impact at hospitals. Further analysis indicated that a greater physician density at PHCs increased the probability of DM patients visiting PHCs relative to village clinics, in contrast with the decreasing likelihood of these patients visiting county hospitals, which is consistent with findings from a study that the introduction of primary care providers would lead to a shift of care from specialists to primary care for diabetes patients and increased the number of primary care visits [35]. Recruitment and allocation of human resources for health in PHCs would lead to higher health care utilization of DM patients.

Another finding from this study that positive impact of physician density on use of outpatient service by DM patients was relatively greater in the areas with low availability of physicians, such as rural and western China, than urban area and other regions, is important for human resource policies. To invest more human resources for health in disadvantaged area has been appealed for long time, but no significant progress is made. Diminished marginal return of increasing health inputs in high-resource-density areas implies better strategies that priorities of health resource allocation need to focus on the resource-poor areas [22]. Unbalanced distribution of health workers is associated with inequality in access to health care in China [34]. PHCs in rural area and westerns region face more challenges than urban and other regions in recruiting and retaining qualified health workers [36, 37]. In urban cities and high-income provinces, medical graduates and health workers try to find jobs in hospitals rather than in PHCs [16].

Findings from this study could lead to two main policy implications. First, availability of health workers should be improved, especially for PHCs and disadvantaged areas, to meet increasing health needs. To narrow the gap between supply and demand of human resource for health, middle and long term national plans for human resource development for health should be developed. Appropriate incentive policies, including increase in incomes and opportunities in professional development, are needed for attracting qualified health workers to work in PHCs and disadvantaged areas. For health care for DM patients, to integrate diabetes care with other chronic disease control programs is ways of addressing shortage of health workers by sharing the human resources. Second, strategies for addressing the maldistributions of health workforce are needed. Since 2011, Chinese government has implemented a special education program targeting the PHCs and remote rural area, in which medical students are financially supported by the government for their studies and they must work in targeted area and facilities for a time period after graduation [38]. Such kind of programs is very important for addressing shortage of health workforce in PHCs and disadvantaged area.

### Study limitations

The results of this study should be interpreted within the context of several limitations. Due to data limitation, several possible confounding factors could not be captured. For example, health insurance copayment that could affect use of outpatient service by DM patients was not included because it was not available in the data. We tried to overcome this problem through application of multilevel regression in which health insurance status and the years 2008 and 2013 were included as dependent variables. In addition, with the complexity of DM and its complications, we were not able to identify the possible influence of concurrent diseases and comorbid conditions on health care seeking behavior [30], even though we used sickbed days trying to reduce residual error as much as possible.

## Conclusion

In conclusion, this study suggested that health workforce is a key factor for improving accessibility to health care. Ensuring an adequate availability of health workforce in PHCs is one of top priorities to improve health care delivery for DM patients. Allocation of human resources for health should focus on primary health facilities in rural and western region.

## Abbreviations

CHC: Community health centers
DM: Diabetes mellitus
GDP: PGDP, per capita
GDP: Gross Domestic Product
IRR: Incidence rate ratio
ML_ZINB: Multilevel zero-inflated negative binomial regression
MML: Multilevel multinomial logit model
NHFPC: National Health and Family Planning Commission
NHSS: China National Health Service Survey
NRCMS: New Rural Cooperative Medical Scheme
OOP: Out-of-pocket payments
PHC: Primary health centers
RMB: Chinese Renmingbi
RRR: Relative risk ratio
THC: Township health centers
UEBMI: Urban Employee-based Medical Insurance
URBMI: Urban Resident-based Health Medical Insurance schemes
